# Below-Ground Growth of Alpine Plants, Not Above-Ground Growth, Is Linked to the Extent of Its Carbon Storage

**DOI:** 10.3390/plants10122680

**Published:** 2021-12-06

**Authors:** Youfu Zhang, Tuo Chen, Hanbo Yun, Chunyan Chen, Yongzhi Liu

**Affiliations:** 1 Department of Biological Science, Henan University of Science and Technology, Luoyang 471003, China; ccy0713@126.com; 2 State Key Laboratory of Cryospheric Sciences, North-West Institute of Eco-Environmental and Resources, Chinese Academy of Sciences, Lanzhou 730000, China; hbyun@lzb.ac.cn (H.Y.); liuyz@lzb.ac.cn (Y.L.)

**Keywords:** Qinghai–Tibetan Plateau, alpine meadow, carbon storage, biomass

## Abstract

Understanding carbon allocation in plants is essential for explaining their growth strategies during environmental adaptation. However, the role of mobile carbon in plant growth and its response to habitat conditions is still disputed. In degraded meadow (alpine sandy grassland) and non-degraded meadow (typical alpine meadow and swamp meadow) on the Qinghai–Tibetan Plateau, we measured the monthly averages of above-ground biomass (AGB) and below-ground biomass (BGB) of the investigated species in each meadow and the average concentration of non-structural carbohydrates (NSCs), an indicator of carbon storage. Below-ground organs had higher concentrations and showed more seasonal variation in NSCs than above-ground organs. BGB had a positive correlation with below-ground NSCs levels. However, AGB had no clear relationship with above-ground NSCs levels. Plants in sandy grasslands had higher total NSC, soluble sugars, fructose, and sucrose concentrations and lower starch concentrations in below-ground organs than plants in alpine or swamp meadows. Overall, NSCs storage, particularly soluble sugars, is a major process underlying the pattern of below-ground growth, but not above-ground growth, in the meadow ecosystem of the Qinghai–Tibetan Plateau, and degraded meadow strengthens this process. These results suggest that the extent of carbon storage in non-photosynthetic organs of alpine herbs impacts their growth and habitat adaptation.

## 1. Introduction

In plant ecology, above- and below-ground biomass allocation is a central issue in understanding how plants adapt to their habitats. Previous studies have explored the patterns of above- and below-ground biomass allocation in response to habitat change [1,2,3,4]. For instance, plants with limited exposure to light have a higher proportion of above-ground biomass to optimize growth and survival [2], whereas they have larger below-ground root systems for better access to soil water/nutrition under conditions of reduced nutrient and/or water supply [3,5]. These observed ecological strategies are well explained by the functional equilibrium hypothesis, which emphasizes that plant strategies are optimized for resource capture when resources are limited [6]. Nevertheless, there is a lack of knowledge on how biomass allocation is regulated to cope with habitat changes.

Photosynthetic carbon is the source of energy and carbon for plant growth. However, the primary processes of photosynthetic carbon metabolism do not explain how plants invest photosynthate to regulate their growth [7,8]. Additionally, there is a continuing debate on whether the availability of fixed carbon constrains plant growth [7,9,10]. Thus, research on the relationship between carbon storage and biomass in different organs can help to reveal the role of carbon in regulating plant growth [10]. The amount of accumulated non-structural carbohydrates (NSCs) is often used as a measure of carbon storage in plants [10,11]. Soluble sugar and starch are the main NSCs. They are strongly related to the instantaneous carbon metabolism demand and long-term carbon storage, respectively. Hence, analyses of the NSC concentration and its subdivided pools of constituents will help understand how the carbon regulation strategy shifts between storage and growth in response to habitat changes.

Alpine habitats have harsh growing conditions with short and cool growing seasons and a short-day photoperiod. Alpine plants usually use large amounts of NSCs for storage rather than growth [12,13]. This utilization pattern helps plants to bridge temporal gaps between carbon supply and demand on a diurnal and seasonal scale [5,14]. However, feedback inhibition of NSCs on photosynthesis easily rises in photosynthetic organs [15]. The roots or rhizomes of non-photosynthetic organs are regarded as a suitable or preferred reservoir for maintaining high NSCs concentrations in perennial plants [2,16]. In turn, the accumulation of NSCs stimulates the growth of roots or rhizomes to meet the demand of increasing reservoir size [10]. Additionally, meadow degradation worsens plant habitats, and the adjustment of NSCs storage may be more important for plants survival in degraded meadows. Therefore, it is assumed that the extent of NSCs storage in non-photosynthetic organs, rather than in photosynthetic organs, is linked to growth and biomass accumulation, especially in a degraded meadow.

To test the above assumption, we examined the relationship between NSCs concentration and biomass and evaluated the role of carbon storage in impacting above- and below-ground growth and its response to the three meadows. The results can help to interpret and assess plant adaptation in meadow desertification.

## 2. Materials and Methods

### 2.1. Study Site and Species

**Figure 1:**
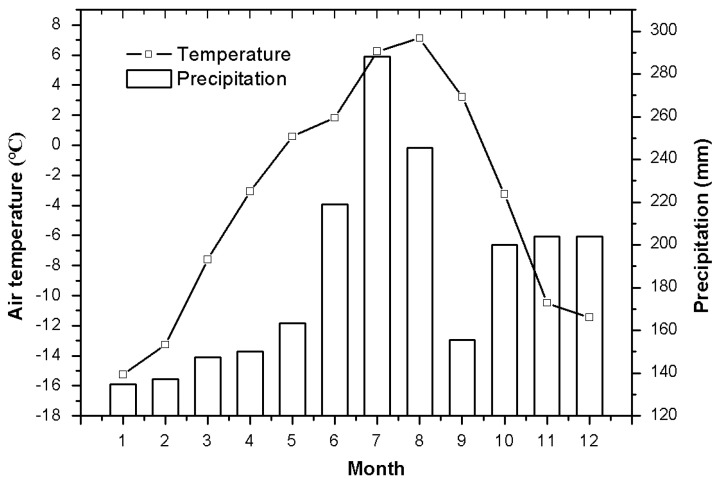
Monthly mean air temperature and precipitation in the investigated area of Qinghai–Tibetan Plateau. Meteorological data from Northwest Institute of Eco-environment and Resources, Chinese Academy of Sciences.

**Figure 2:**
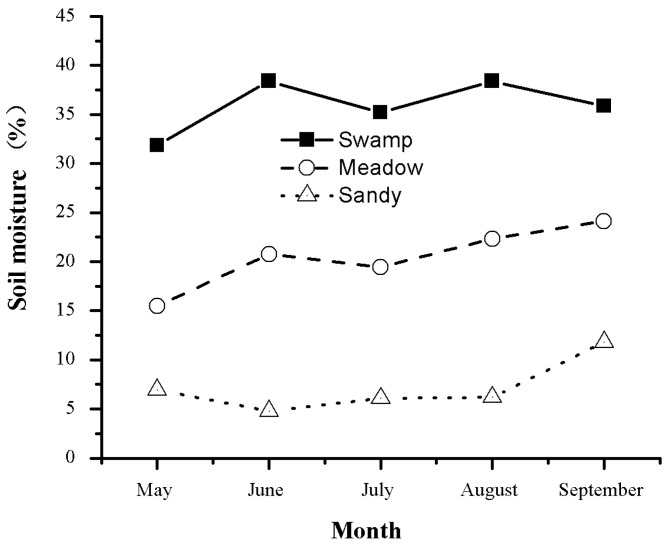
Seasonal variations in soil moisture during growing periods in the meadows.

With a mean elevation of 4000 m a.s.l., the Qinghai–Tibetan Plateau has a unique climate and minimal anthropogenic influences. It is regarded as an ideal place to study the relationship between plants and alpine habitats [17]. Over 60% of the plateau is covered by meadows. Alpine meadow ecosystems are very fragile and sensitive to the climate. The study area is located at 34°51.26′ N and 92°56.35′ E, with an elevation of 4628 m a.s.l. on the Qinghai–Tibetan Plateau, China. The area has a typical continental highland climate. Mean monthly air temperature and precipitation were taken from the Beiluhe experimental station record in 2012 (Figure 1). Natural alpine meadow ecosystems were categorized as typical alpine meadows, alpine sandy grasslands, and swamp meadows. The investigated plants in the three studied meadows are listed in Table 1. A mixed soil sample was collected at 0–60 cm depth from 3–4 sites on the day of sampling. Soil moisture was measured by the gravimetric method and expressed as a percentage of dry soil mass (Figure 2).

### 2.2. Sampling and Analysis of NSCs

Samples were collected according to the methods of Yang [18]. The plants were sampled in the last 7 days of each month from May to September in 2012 in three alpine meadows (Table 1). Except for *Poa pratensis*, species collected in typical alpine meadows and alpine sandy grasslands are perennial and have rhizomes. The same species were collected in alpine sandy grassland and typical alpine meadows, which is advantageous for comparing differences in communities between degraded and non-degraded meadows. However, species in swamp meadows were different from those in the other two meadows. Samples were taken around noon on a clear day. On each sampling occasion, eight sites at intervals of 50 m were randomly selected in each meadow. The collected plants were separated from a 25 cm × 25 cm soil cylinder with a depth of approximately 40 cm using a spade. The sampled species were collected (some fine roots may have been lost) and cleaned, followed by absorption of excess water by absorbent paper. The same species from different sites were counted in each meadow (Table 1), separated into above- and below-ground parts, and transferred into envelopes. They were dried in a 105 °C oven for 15 min and then in a 70 °C oven for 48 h, followed by weighing. The dried samples were ground into fine powder through a 0.5 mm sieve for further analysis.

An accurately weighed 0.100 g ground sample was soaked in 80% ethanol, placed at 80 °C in a water bath for 30 min, and centrifuged at a speed of 4000 rpm for 20 min, and then the supernatant was collected. The repeated extraction process was performed three times for each sample [16]. The supernatants of the three extractions were combined and then diluted to 50 mL with 80% ethanol as the soluble sugar extraction. Total soluble sugars, fructose, and sucrose were measured by the anthrone method in a spectrophotometer at 620 nm following the procedure of Kang with minor adjustments [19]. Briefly, 0.1 mL of the extraction was mixed with 5 mL of anthrone reagent (100 mg of anthrone in 100 mL of 70% (*v*/*v*) H_2_SO_4_) and incubated at 90 °C for 15 min to measure total soluble sugars. A 0.1 mL of the extraction was mixed with 0.1 mL of 7.6 mol·L^−1^ KOH at 100 °C for 10 min, and then the anthrone reagent was added and incubated at 40 °C for 15 min to measure sucrose. A 0.5 mL of the extraction was evaporated to 0.1 mL; the chilled anthrone reagent was added and incubated at 25 °C for 90 min to measure fructose. The residue was used to extract starch after hydrolysis with 52% perchloric acid. The concentration of starch was determined in a spectrophotometer at 620 nm after using the anthrone reagent [19,20]. Starch plus total soluble sugars were defined as total NSC.

### 2.3. Statistical Analysis

The relationships of total NSC, soluble sugars, fructose, sucrose, and starch concentrations with the above- and below-ground biomass were determined by Spearman correlation analysis in SPSS 11.5 for Windows. The effects of the type of meadow, season, and organ on the concentrations of total NSC and its components were determined by ANOVA analysis in SPSS 11.5. Linear regressions were used to evaluate associations of NSCs concentrations with above- and below-ground biomass by using the software Origin 7.0.

## 3. Results

### 3.1. Seasonal Variation in Vegetation Biomass

**Figure 3:**
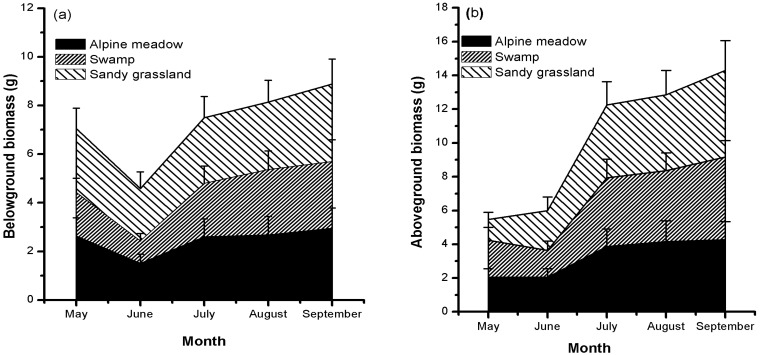
Seasonal variations in mean dry below-ground (**a**) and above-ground (**b**) biomass in the three meadows. Each value is the monthly mean of the individual biomass of all species collected in each meadow.

The mean biomass of above- and below-ground organs generally increased from June to September in all meadows. However, from May to June, the below-ground biomass (BGB) all distinctly decreased, and above-ground biomass (AGB) had different trends in the three meadows (Figure 3). Sandy grassland had the highest AGB and BGB in all meadows from June to September; thus, biomass accumulation increased in degraded grassland.

### 3.2. Relationships between Biomass and NSCs in Above- and Below-Ground Organs

**Figure 4:**
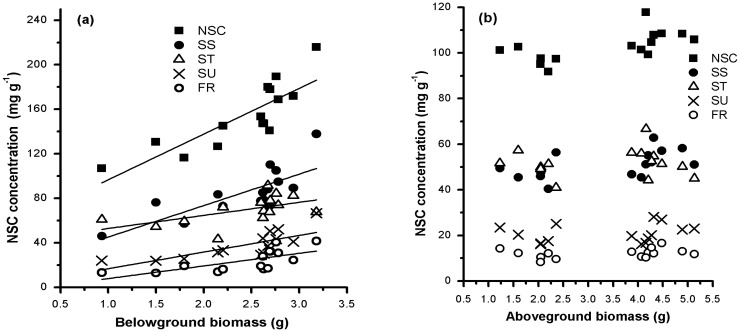
Mean below-ground (**a**) and above-ground (**b**) biomass related to non-structural carbohydrates (NSCs) concentrations. Each value is the monthly mean of individual biomass and NSCs of all species collected in each meadow.

BGB was positively correlated with the concentrations of total NSC and its components (Figure 4a). Their linear regression equations are as follows: total NSC (y = 41.004x + 55.687, R^2^ = 0.671, *p* < 0.001), soluble sugars (y = 28.261x + 16.936, R^2^ = 0.563, *p* < 0.001), fructose (y = 11.349x − 3.503, R^2^ = 0.440, *p* < 0.01), sucrose (y = 14.948x + 1.758, R^2^ = 0.520, *p* < 0.01), and starch (y = 41.1567 + 11.74417x, R^2^ = 0.265, *p* > 0.05). However, there were no clear relationships between AGB and NSCs concentrations (Figure 4b). Therefore, enhanced NSCs storage can promote below-ground growth but not above-ground growth. Moreover, soluble sugars seem to be more important than starch in this process.

### 3.3. Seasonal Variance Features of NSCs in Above- and Below-Ground Organs

In the three meadows, the coefficients of seasonal variance of total NSC, soluble sugars, fructose, sucrose, and starch concentrations were all higher in below-ground organs than in above-ground organs (Table 2). These results show that the storage of below-ground NSCs had a larger seasonal variation and displayed more sensitivity to variance in growth season than the storage of above-ground NSCs.

The correlation coefficients of total NSC with below-ground and above-ground biomass were all positive among the investigated meadows (*p* < 0.05) (Table 2). However, their correlation coefficients of soluble sugar and starch were distinctly different among the three meadows. In particular, the correlation coefficient of soluble sugars was positive in typical alpine meadows (*p* < 0.01) and swamp meadows (*p* < 0.05) and negative in sandy grasslands (*p* > 0.05). The correlation coefficient of starch was positive in typical alpine meadows and sandy grasslands (*p* < 0.01) and negative in swamp meadows (*p* > 0.05). These results reflect differential partitioning of soluble sugar and starch storage between AGB and BGB among the three meadows, which is likely linked to their physiological adaptation to various conditions among meadow habitats.

### 3.4. Differences in NSCs Concentrations in Above- and Below-Ground Organs

The plants investigated in sandy grassland had higher total NSC concentrations, with higher soluble sugar, fructose, and sucrose concentrations and lower starch concentration than those in the alpine or swamp meadow (Table 3). The plants in sandy grassland also had the highest below-ground–above-ground ratio of total NSC, soluble sugars, fructose, and sucrose among the three meadows (Table 3). Therefore, degraded meadows, namely, sandy grasslands, allocate more NSC storage to below-ground than both non-degraded meadows.

Variations in the type of meadow, the organ (above-and below-ground organs) and seasonality (different months) had various impacts on the concentrations of total NSC and NSC components (Table 3). The organ difference all impacted total NSC, soluble sugar, sucrose, fructose, and starch concentrations (*p* < 0.001). In contrast to the season, the meadow type impacted the concentrations of soluble sugars (*p* < 0.05) and starch (*p* > 0.05). A significant interaction between season and organ was found for total NSC (*p* < 0.05) but not for other NSCs components (*p* > 0.05) (Table 4). These results indicate that the change in total NSC storage primarily relies on the levels of soluble sugar and starch, which change in response to variation in the meadow type and season, respectively.

## 4. Discussion

In the alpine meadows on the Qinghai–Tibetan Plateau, we explored the relationship between NSC storage and biomass accumulation, and we examined whether the relationship differs between below-ground and above-ground parts. BGB was positively correlated with below-ground NSCs concentrations (*p* < 0.05). However, AGB had no clear relationship with above-ground NSCs concentrations (Figure 3). This difference can be attributed to the prioritization of below-ground carbon storage in the mountain environment [13,21] or perennial grasses [2]. Moreover, high mobile carbon storage can promote cell division and growth [22] and increase the carbon reservoir. Compared with below-ground growth, above-ground growth is not directly related to the self-contained carbon storage, since the excess carbon will flow to roots or other storage organs for storage during above-ground growth limitation. Hence, it is reasonable to believe that the carbon surplus caused by the limited growth of photosynthetic organs, in turn, promotes the growth of storage organs in harsh alpine conditions. This finding supports observations in previous studies, such as increased below-ground biomass in low-temperature conditions [5,18,23,24], increased root/shoot ratios with elevation [25,26], and dwarf stature of alpine plants [13]. In summary, the results of this study show that below-ground carbon storage is a growth-promoting factor for the below-ground organs of alpine plants.

NSCs storage can be subdivided into starch and soluble sugar reservoirs. Interestingly, the concentrations of soluble sugars rather than starch had a strong positive correlation with BGB. This suggests that the extent of soluble sugar storage, rather than starch storage, is directly linked to below-ground growth in alpine plants. Although starch in photosynthetic organs is usually remobilized at night to support respiration and growth during diurnal cycles [22], starch is not usually used for growth metabolism unless soluble sugar is exhausted in storage organs. Starch is regarded as a regulator of plant growth and soluble sugar storage [27]. Thus, the positive correlation between BGB and the concentration of soluble sugars is readily maintained below-ground. This result suggests that soluble sugars rather than starch are more likely to affect below-ground growth.

Meadow degradation can promote an increase in below-ground carbon storage and biomass accumulation in alpine plants. In this investigation, sandy grasslands had higher BGB and concentrations of soluble sugars than typical alpine meadows or swamp meadows. This may be related to the variation in soil texture. In sandy grasslands, increased soluble sugars can provide a source of immediate energy supply for the increased metabolic demand of new root growth. Moreover, loose sand allows deeper penetration of roots, and plants can use deep soil nutrients and water. This helps to explain why the highest below-ground biomass was observed in alpine sandy grasslands (Table 3), and also provides evidence for the functional equilibrium hypothesis [6]. A similar result was also found in another study on the Qinghai–Tibet Plateau [16]. It can be inferred that the variation in soil texture caused by meadow degradation changes the carbon demand for below-ground growth in alpine meadows, which is considered a driver of below-ground carbon allocation. Additionally, compared with typical alpine meadows and swamp meadows, sandy grasslands with reduced soil moisture (Figure 2) and vegetation coverage have lower soil temperature and higher variability in soil temperature during the freezing-thawing process [28,29]. In this case, soluble sugars rather than starch can be used as an osmotic regulator to protect plants from cold temperatures [30]. Moreover, increased soluble sugars have also been observed in cold-treated plants [31]. These results suggest that meadow degradation can change carbohydrate allocation according to the physiological demand and consequently cause changes in biomass accumulation.

Below-ground carbohydrate storage of perennial plants needs to be remobilized upon the initiation of growth. As expected, lower below-ground biomass (Figure 3) and NSC concentrations were observed during the growth period (May) and afterwards (June). This may be related to cooling conditions in June (Figure 2). Similar results were found in alpine plants growing in Wyoming [12] and the Alps [13]. This result further verifies the necessity of high below-ground biomass in alpine meadows [24]. However, this conflicts with previous results in temperate grasslands [32], which indicated that higher below-ground biomass and its ratio occurred under warming rather than cooling conditions. Because climate warming will increase the water demand of plants through evapotranspiration, increased roots can enable plants to absorb more soil water [32]. These differences may be related to changes in the temperature response range of local species [33]. However, for the same species investigated in typical alpine meadows and alpine sandy grasslands, our results suggest that increased below-ground carbon storage is related to low temperature in different alpine meadows.

## 5. Conclusions

NSCs storage is a necessary process to regulate the carbon supply for plant growth and metabolism. The statistically positive correlation between BGB and NSCs levels shows that the below-ground NSC storage has a positive effect on its growth. Moreover, meadow degradation due to desertification increases the total NSC and soluble sugar storage and below-ground growth, which enhances carbon supply and security in a harsh habitat. Thus, increasing carbon storage in below-ground organs can be regarded as an adaptation to degraded meadows. However, above-ground NSCs storage does not directly reflect the physiological carbon supply for growth itself. Overall, below-ground growth, rather than above-ground growth, is linked to the extent of carbon storage itself for alpine plants. It can be inferred that the functional traits of below-ground organs may be more appropriate for explaining the variation in community productivity in different meadows.

## Figures and Tables

**Table 1:** Species sampled and number of samples in the three investigated meadows.

Type of Meadow	Species
Alpine sandy grassland	*Carex moorcroftii* Falc. ex Boott (17), *Astragalus mongholicus* Bunge (13), *Leontopodium alpinum* Cass (40), *Androsace tapete* Maxim (15), *Aster tongolensis* Franch (21), *Kobresia pygmaea* C. B. Clarke (23), *Microula sikkimensis* (Clarke) Hems (9), *Salvia prionitis* Hance (33), *Poa annua* L. (12)
Typical alpine meadow	Same species listed as in alpine sandy grassland, the number of the species is 21, 23, 40, 15, 17, 14, 7, 45, 13, respectively.
Swamp meadow	*Carex moorcroftii* Falc. ex Boott (13), *Astragalus mongholicus* Bunge (13)*, Cremanthodium humile* Maxim (8), *Delphinium grandiflorum* L. (13), *Allium carolinianum* DC. (7)*, Pedicularis tibetica* Franch (14), *Saussurea superba* J. Anthony (20), *Polygonum viviparum* L. (7)*, Polygonum sibiricum* Laxm (20)

Note: the average number of individuals of species sampled in different months is shown in parentheses or listed.

**Table 2:** Coefficients of seasonal variance and correlation of NSCs with above- and below-ground biomass.

Variables	Typical Alpine Meadow			Swamp Meadow			Alpine Sandy Grassland		
	Below	Above	Correlation	Below	Above	Correlation	Below	Above	Correlation
NSC	12.53	8.53	0.901 **	22.53	5.99	0.681 *	20.08	4.56	0.725 **
SS	7.64	5.58	0.994 **	31.44	15.20	0.798 *	22.03	9.56	−0.049
ST	18.90	12.99	0.818 **	14.59	9.99	−0.211	24.28	12.08	0.832 **
SU	21.10	10.68	0.484 *	23.99	13.12	0.450 *	26.17	8.58	−0.171
FR	23.72	21.50	0.978 **	48.02	7.37	0.694 *	34.40	20.60	0.363

NSC, total non-structural carbohydrate; SS, soluble sugars; ST, starch; SU, sucrose; FR, fructose. Above and below represent coefficients of seasonal variance for parameters in the above- and below-ground organs, respectively; correlation represents the correlation coefficient of parameters with below-ground and above-ground biomass, * and ** indicate that the correlation is significant at the 0.05 and 0.01 levels, respectively.

**Table 3:** The mean values and ratios of NSCs in the above- and below-ground organs.

Variables	Typical Alpine Meadow			Swamp Meadow			Alpine Sandy Grassland		
	Below	Above	Ratio	Below	Above	Ratio	Below	Above	Ratio
NSC	156.6 ± 19.6	103.6 ± 8.8	1.50 ± 0.07	139.7 ± 32.1	100.7 ± 6.0	1.38 ± 0.18	167.3 ± 33.6	104.2 ± 4.7	1.59 ± 0.19
SS	82.0 ± 6.3	48.7 ± 2.7	1.68 ± 0.03	70.8 ± 22.3	49.0 ± 7.4	1.43 ± 0.21	102.3 ± 22.5	55.4 ± 5.3	1.86 ± 0.33
ST	74.6 ± 14.1	54.9 ± 7.1	1.35 ± 0.11	68.8 ± 10.0	51.8 ± 5.1	1.34 ± 0.18	65.0 ± 15.6	48.8 ± 6.0	1.32 ± 0.13
SU	32.3 ± 6.8	17.9 ± 1.9	1.79 ± 0.15	32.1 ± 7.7	19.1 ± 2.5	1.69 ± 0.25	48.9 ± 12.0	25.3 ± 2.1	1.95 ± 0.42
FR	18.0 ± 4.3	11.3 ± 2.4	1.58 ± 0.05	24.3 ± 11.6	12.1 ± 0.9	1.97 ± 0.59	29.3 ± 10.0	12.9 ± 2.7	2.28 ± 0.57

Values are the means (mg g^−1^) ± SE; Ratio is below-ground–above-ground; NSC, total non-structural carbohydrate; SS, soluble sugars; ST, starch; SU, sucrose; FR, fructose.

**Table 4:** The effects of type of meadow, season, and organ on carbohydrate compositions.

	Meadow (M)	Season (S)	Organ (O)	Interactions		
				(M-O)	(S-O)	(M-S)
NSC	ns	***	***	ns	**	ns
Soluble sugars	*	ns	***	ns	ns	ns
Starch	ns	**	***	ns	ns	ns
Sucrose	***	ns	***	ns	ns	ns
Fructose	ns	**	***	ns	ns	ns

Based on a hierarchical ANOVA analysis. *** *p* < 0.001; ** *p* < 0.01; * *p* < 0.05; ns *p* ≥ 0.05. Meadow represents three types of meadows. Season represents different months. Organ represents above-ground and below-ground plant parts.

## Data Availability

Data is contained within the article.
